# Acute respiratory infections, influenza-like illness and JIA: impact on disease activity and response to the influenza vaccine

**DOI:** 10.1186/1546-0096-10-S1-A103

**Published:** 2012-07-13

**Authors:** Luciana M Carvalho, Flávia E Paula, Rodrigo VD Silvestre, Luciana R Roberti, Wyller A Mello, Eurico Arruda, Virginia PL Ferriani

**Affiliations:** 1 Evandro Chagas Institute (WHO National Influenza Center), Ananindeua, Para, Brazil; 2 University of São Paulo Ribeirão Preto, Ribeirao Preto, Sao Paulo, Brazil

## Purpose

To evaluate the frequency of ARI and IL in JIA patients and its impact on disease activity, and to assess immunogenicity, safety and effectiveness of Flu vaccine in JIA patients using immunosuppressive drugs, including anti-TNFα.

## Methods

Surveillance for Flu and other respiratory virus: respiratory syncytial virus (RSV), metapneumovirus (MPV), parainfluenza virus (PIV), bocavirus (Bov), adenovirus (Adv), rhinovirus (RV) and coronavirus (Cov) was conducted in JIA patients attending a tertiary Pediatric Rheumatology Clinic from March to August 2007 (61 JIA patients included) and from March to August 2008 (63 JIA patients). Patients presenting signs of ARI and/or IL had airway secretion collected in a 72hs period, by combined nasal and pharyngeal swab, and viruses detection was performed using real time PCR (qPCR). 44 JIA patients aged 2 to 18 years received trivalent split Flu vaccine A/Solomon Islands/3/2006, H1N1; A/Brisbane/10/200, H3N2; B/Florida/2006 (Sanofi Pasteur SA/ Butantan Institute) in 2008. Thirty-one of the patients (70%) were on MTX or leflunomide and 5 (11%) were on anti-TNF drugs at the time of vaccination. Clinical and laboratory evaluation, including ACRPed30 were performed during the surveillance period, before and after vaccination. Flu titers were determined by hemagglutination inhibition (HI) assay before and 30-40 days after vaccination. Immune response was defined by seroconversion (a 4-fold or greater rise in HI antibodies) and seroprotection (HI titers at least 1:40).

## Results

During the surveillance period, 105 ARI episodes were reported by the patients, and 28 of these episodes (26,6%) were IL. Of 33 samples collected, 20 (60%) were positive for at least one respiratory virus: Flu A 25%, Flu B 5%, RV 30%, Adv 20%, PIV 15%, RSV 10%, Bov 5%, HCov 5%. Virus co-infections were detected in 15% of the positive samples. During the same period, 49 JIA flares were observed and 8 (16%) had a temporal relationship with ARI without any other plausible cause that may have triggered the events. Seroprotection rates after vaccination were higher than 70% (91-100%) for all 3 Flu strains and seroconversion rates exceeded 40% (74-93%). In general, response to Flu vaccine was not influenced by therapeutic regimens or disease activity, but patients using anti-TNFα drugs presented lower response to H1N1 strain. No significant differences were found in JIA activity index (*ACRPed30*) after vaccination and no patient reported IL symptoms during the 6-month post-vaccine period.

## Conclusion

ARI are relatively frequent in JIA patients and can possibly have a role in JIA flares. Trivalent split FLU vaccine seems to be immunogenic, safe and effective in JIA patients using immunosuppressive agents and should be given to all children with JIA.

## Disclosure

Luciana M. Carvalho: None; Flávia E. Paula: None; Rodrigo V.D. Silvestre: None; Luciana R. Roberti: None; Wyller A. Mello: None; Eurico Arruda: None; Virginia P.L. Ferriani: None.

